# Supplementary material for: Immune suppression is associated with enhanced systemic inflammatory, endothelial and procoagulant responses in critically ill patients
Source: PLoS One. 2022 Jul 25;17(7):e0271637. doi: 10.1371/journal.pone.0271637 (PMC9312372; doi:10.1371/journal.pone.0271637)
Supplement: S1 Table — Abbreviations: COPD, chronic obstructive pulmonary disease. (DOCX) [file pone.0271637.s001.docx]

S1 Table

| Admission diagnosis | n |
| --- | --- |
| Sepsis admission diagnoses | 51 |
| Community-acquired pneumonia | 27 |
| Peritonitis | 7 |
| Hospital-acquired pneumonia | 4 |
| Necrotizing fasciitis | 3 |
| Urinary tract infection | 5 |
| Mediastinitis | 1 |
| Brain abscess | 1 |
| Primary meningitis | 1 |
| Pharyngitis | 1 |
| Sinusitis | 1 |
| Noninfectious admission diagnoses | 26 |
| Cardiac arrest | 6 |
| Subdural hematoma / intracranial hemorrhage | 5 |
| Cerebrovascular accident / stroke | 3 |
| Cardiogenic shock | 2 |
| Exacerbation of COPD | 2 |
| Anaphylaxis | 1 |
| Asthma | 1 |
| Coma | 1 |
| Gastrointestinal ischemia | 1 |
| Cardiomyopathy | 1 |
| Gastrointestinal bleeding | 1 |
| Pulmonary hemorrhage / hemoptysis | 1 |
| Thoracic aortic aneurysm dissection | 1 |
