# Supplementary figures and images for: Immune suppression is associated with enhanced systemic inflammatory, endothelial and procoagulant responses in critically ill patients
Source: PLoS One. 2022 Jul 25;17(7):e0271637. doi: 10.1371/journal.pone.0271637 (PMC9312372; doi:10.1371/journal.pone.0271637)

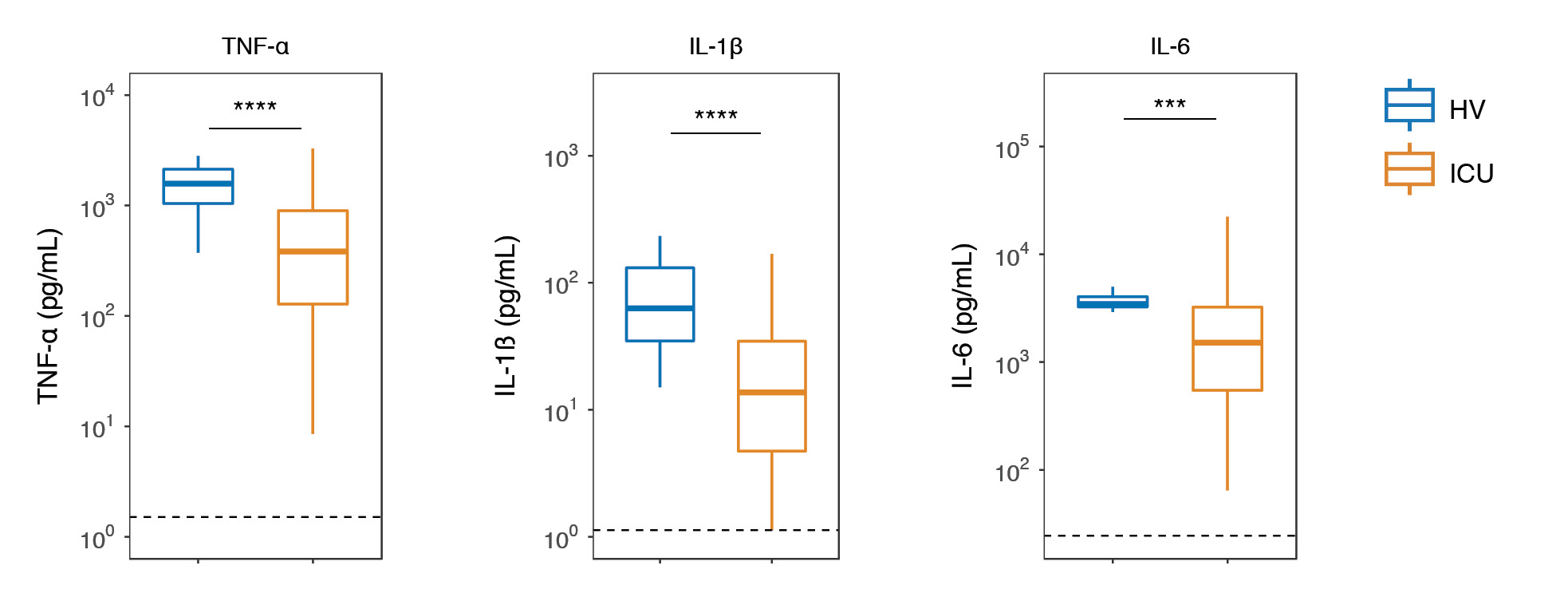

Supplement: S1 Fig — Whole blood was drawn from 77 critically ill patients at 9:00 AM on the first day after admission to the ICU and from 19 age- and sex-matched healthy controls. Blood was stimulated for 3 hours with ultrapure LPS (100 ng/mL), and tumor necrosis factor (TNF)-α and interleukin (IL)-1β, and IL-6 concentrations were measured in supernatants. Data are presented box and whisker diagrams as specified by Tukey. HV, healthy volunteers; ICU, critically ill patients. ***P < 0.001, ****P < 0.0001. (TIF) [file pone.0271637.s002.tif]

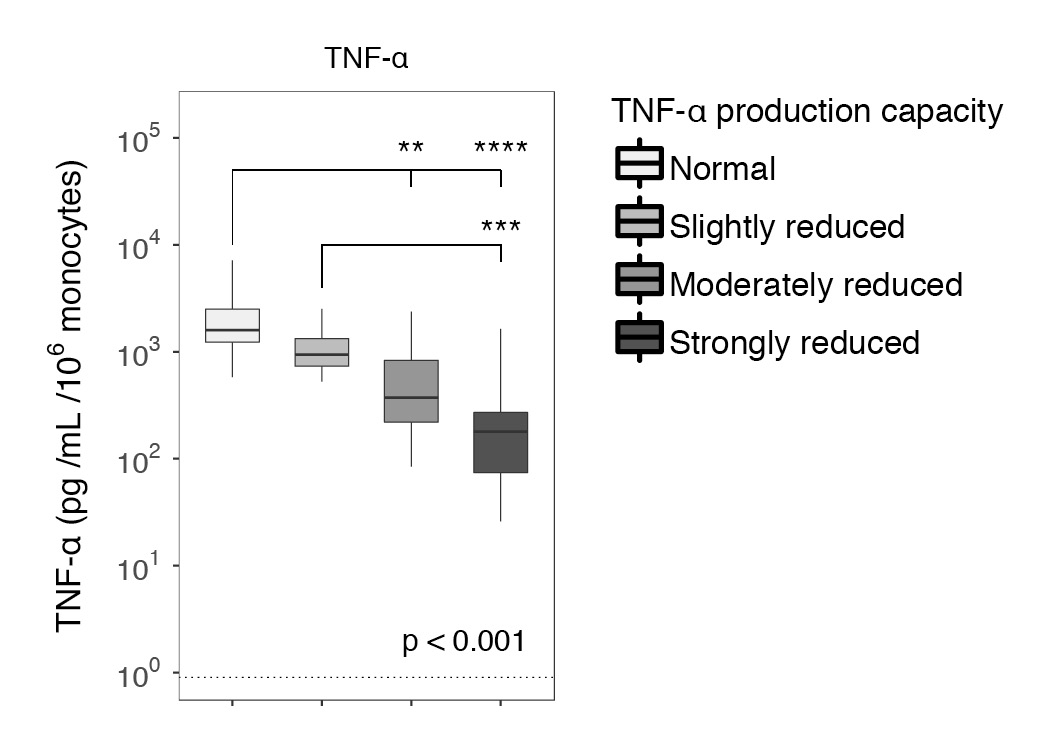

Supplement: S2 Fig — Whole blood from critically ill was stimulated for 3 hours with ultrapure LPS (100 ng/mL). Tumor necrosis factor (TNF)-α concentration was measured in supernatants. TNF-α concentrations per 106 monocytes in whole blood are stratified according whole blood TNF-α production capacity (i.e. quartiles of TNF concentration in supernatants after LPS stimulation). Data are presented as box and whisker diagrams as specified by Tukey, in 59 patients in whom white blood cell differentials were available. ** P < 0.01, ***P < 0.001, ****P < 0.0001. (TIF) [file pone.0271637.s003.tif]
