# Supplementary material for: Immune suppression is associated with enhanced systemic inflammatory, endothelial and procoagulant responses in critically ill patients
Source: PLoS One. 2022 Jul 25;17(7):e0271637. doi: 10.1371/journal.pone.0271637 (PMC9312372; doi:10.1371/journal.pone.0271637)
Supplement: Attachment — Submitted filename: Response to Reviewers.docx [file pone.0271637.s006.docx]

­­

To the editorial office of PLOS ONE

|  | Department of Medicine  Center for Experimental Molecular Medicine  Prof. Dr. T. van der Poll, MD, PhD  Phone: +31-20-566 9111  Email: t.vanderpoll@amsterdamumc.nl |
| --- | --- |
|  |  |

Date:	May 7^th^ , 2022

Subject:	Resubmission of manuscript

Dear Dr. Ehab Farag,

With pleasure we hereby submit our revision of the manuscript “Immune suppression is associated with enhanced systemic inflammatory, endothelial and procoagulant responses in critically ill patients".

The manuscript adheres to *PLOS ONE* guidelines: title, abstract and manuscript do not exceed word limits, and our point-by-point responses to the editors and reviewers’ comments are attached to this letter and highlighted in the manuscript. We added two affiliations since one of the co-authors recently moved to another faculty. This is highlighted in the manuscript.

We look forward to your reaction and hope you find our manuscript suitable for publication in *PLOS ONE.*

Sincerely yours, on behalf of all coauthors,

Xanthe Brands, MD

Fabrice Uhel, MD

Tom van der Poll, MD

Attachment: point-by-point responses to comments of the editor and reviewers

**Point-by-point responses to comments of the editor and reviewers**

We would like to gratefully thank the editor and reviewer for carefully evaluating our manuscript. We could address all issues and concerns that were raised and here provide a point-by-point response to each of them:

**Comments from the reviewer:**

**Reviewer** This manuscript investigates immune biomarkers in plasma correlated with inflammatory pathways using patients admitted to ICU. The statistical analysis methods are fine for the presented data analysis. I have below questions and comments.

Comment #1: The objective of this study was to determine the relationship between the degree of immune suppression and systemic inflammation in patients shortly after admission to the ICU. Was the TNF-a used to identify the degree of immune suppression?

*Response:*

*We thank the reviewer for his/her insightful comments.*

*Indeed, as specified in the results section (page 7, lines 143-146, 152-153): “Given that a reduced TNF-α production capacity by blood leukocytes has been widely recognized as a hallmark of immune suppression in critically ill patients, we stratified patients into four groups based on quartiles of LPS-induced TNF-α production. […] the stratification of patients based on TNF-α production capacity of blood leucocytes resulted in conditions of increasing degrees of immune suppression.”*

*In other words, the identification of immune suppression was based on a reduced ability of blood leukocytes to produce TNF-α in response to LPS-stimulation.*

*In order to further clarify this readout, we rephrased the objective of the study in the introduction:*

*Introduction (Pages 3, 4): “To this end, we used the decreased capacity of whole blood leukocytes to produce TNF-α in response to LPS-stimulation as a readout for critically-ill patient immune suppression”*

Comment #2: It is an observational study, but it is not clear how the sample size was decided. Please provide sample size considerations.

*Response:*

*We did not perform a formal sample size calculation prior to this study, since to the best of our knowledge previous studies testing the association between whole blood leukocyte stimulations to biomarkers of systemic inflammation have not been performed. Thus, our study provides preliminary estimates of variance, deviations etc.. for the design of future specific studies. This is now commented upon in the statistical paragraph as follows:*

*Methods (page 6): “A formal sample size calculation was not done prior to the study (to the best of our knowledge previous studies associating whole blood leukocyte stimulations with biomarkers of systemic inflammation have not been performed)”*

Comment #3: Table 1 reported the distribution of the stratification TNF-a across infectious diseases. What’s the distribution of the stratification TNF-a across noninfectious diseases? How many noninfectious diseases also have signs of immune suppression?

*Response:*

*Table one shows the distriubution of TNF-α production capacity in all patients admitted to the ICU, including patients admitted for an infectious(n=51) and for a non-infectious diagnosis (n=26). Of these, 40 (78.4%) patients with a sepsis admission diagnosis and 18 (69.2%) patients with a non-infectious admission diagnosis showed reduced TNF production capacity (≤896 pg/mL), consistent with immune suppression.*

*In order to clarify this point, we added the following to the manuscript:*

*Results (page 8)****:*** *“Among patients admitted for a sepsis (n= 51) or for a non-infectious diagnosis (n= 26), 40 (78.4%) and 18 (69.2%) showed reduced TNF production capacity (≤896 pg/mL), respectively.”*

Comment #4: This study examined the correlations between TNF-a and other immune biomarkers by comparing immune biomarkers among quartile stratified group of TNF-a. Spearman’s r was also used for a couple of markers. For these correlations, would patient’s characteristics (e.g. age, sex, BMI, etc.) mediate the relationship between TNF-a and other immune biomarkers?

*Response:*

*As shown in table 1, no association was found between TNF-α production capacity and patient age, sex or BMI. We therefore considered it unlikely that these clinical parameters mediate the association between TNF-α production capacity and other plasma biomarker concentrations.*

**Comments from the editor:**

Comment #1: Please ensure that your manuscript meets PLOS ONE's style requirements, including those for file naming. The PLOS ONE style templates can be found at

https://journals.plos.org/plosone/s/file?id=wjVg/PLOSOne_formatting_sample_main_body.pdf and

https://journals.plos.org/plosone/s/file?id=ba62/PLOSOne_formatting_sample_title_authors_affiliations.pdf.

*Response:*

*The manuscript is adjusted accordingly.*

*Please note that we added two affiliations since one of the co-authors recently moved to another faculty. This is highlighted in the manuscript.*

*Title page (Page 1)*

*7.	Department of Applied Biomedical Science, Faculty of Health Sciences, Mater Dei hospital, University of Malta, Malta.*

*8. Centre for Molecular Medicine and Biobanking, University of Malta, Malta*

Comment #2: We noted in your submission details that a portion of your manuscript may have been presented or published elsewhere. Please clarify whether this publication was peer-reviewed and formally published. If this work was previously peer-reviewed and published, in the cover letter please provide the reason that this work does not constitute dual publication and should be included in the current manuscript

*Response*

*Part of this work is presented during the French Intensive Care Society International Congress 2021 by one of the authors. Flash communication and the abstract has been published online. Proceedings of Reanimation 2021, the French Intensive Care Society International Congress. Ann Intensive Care. 2021;11:97. Available from: https://doi.org/10.1186/s13613-021-00862-0*

*Preliminary results of the current work have been presented as a brief communication during the congress of the French Intensive Care Society International Congress 2021. The abstract of this presentation has been published in the proceedings of the Congress. However, this does not constitute a dual publication given that only the abstract containing preliminary results has been published. The current publication include the extensive final analysis, including figures, tables, and discussion.*

*In order to clarify this point, the following has been added to the manuscript:*

*Introduction (page 4): Part of this work has been presented during the French Intensive Care Society International Congress 2021 [13].*

Comment #3: Please amend either the abstract on the online submission form (via Edit Submission) or the abstract in the manuscript so that they are identical..

*Response:*

*We uploaded the abstract on the online submission form, identical as the abstract in the manuscript*

Comment #4: Please review your reference list to ensure that it is complete and correct. If you have cited papers that have been retracted, please include the rationale for doing so in the manuscript text, or remove these references and replace them with relevant current references. Any changes to the reference list should be mentioned in the rebuttal letter that accompanies your revised manuscript. If you need to cite a retracted article, indicate the article’s retracted status in the References list and also include a citation and full reference for the retraction notice.

*Response:*

*We reviewed the reference list and added the following reference to the manuscript:*

*References (page 16): 13. Proceedings of Reanimation 2021, the French Intensive Care Society International Congress. Ann Intensive Care. 2021;11:97. Available from: https://doi.org/10.1186/s13613-021-00862-0*
